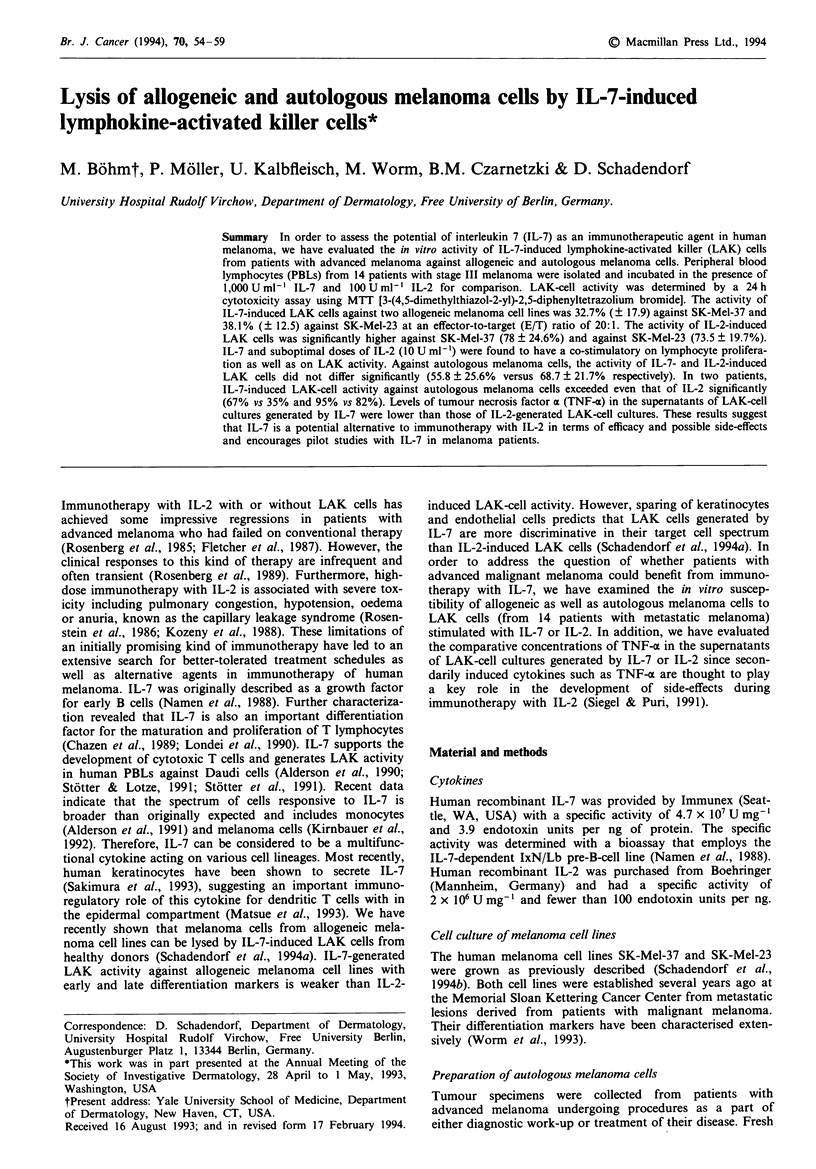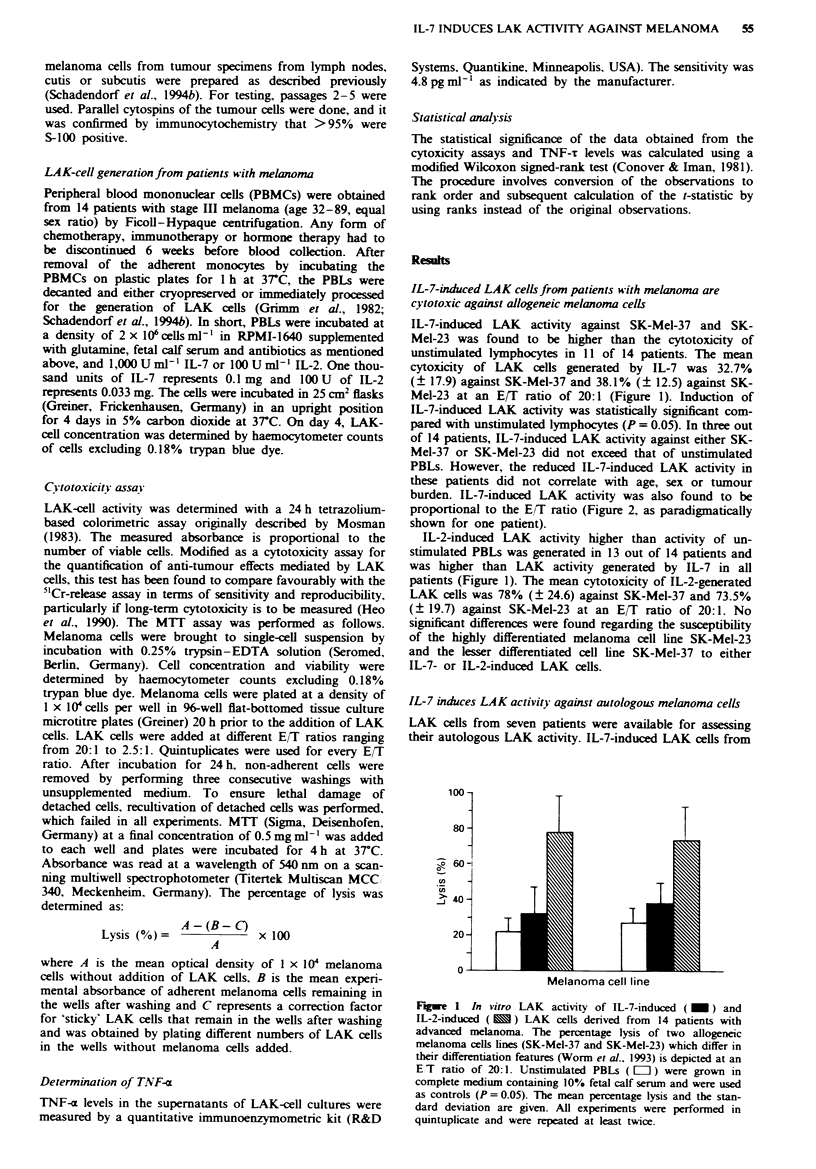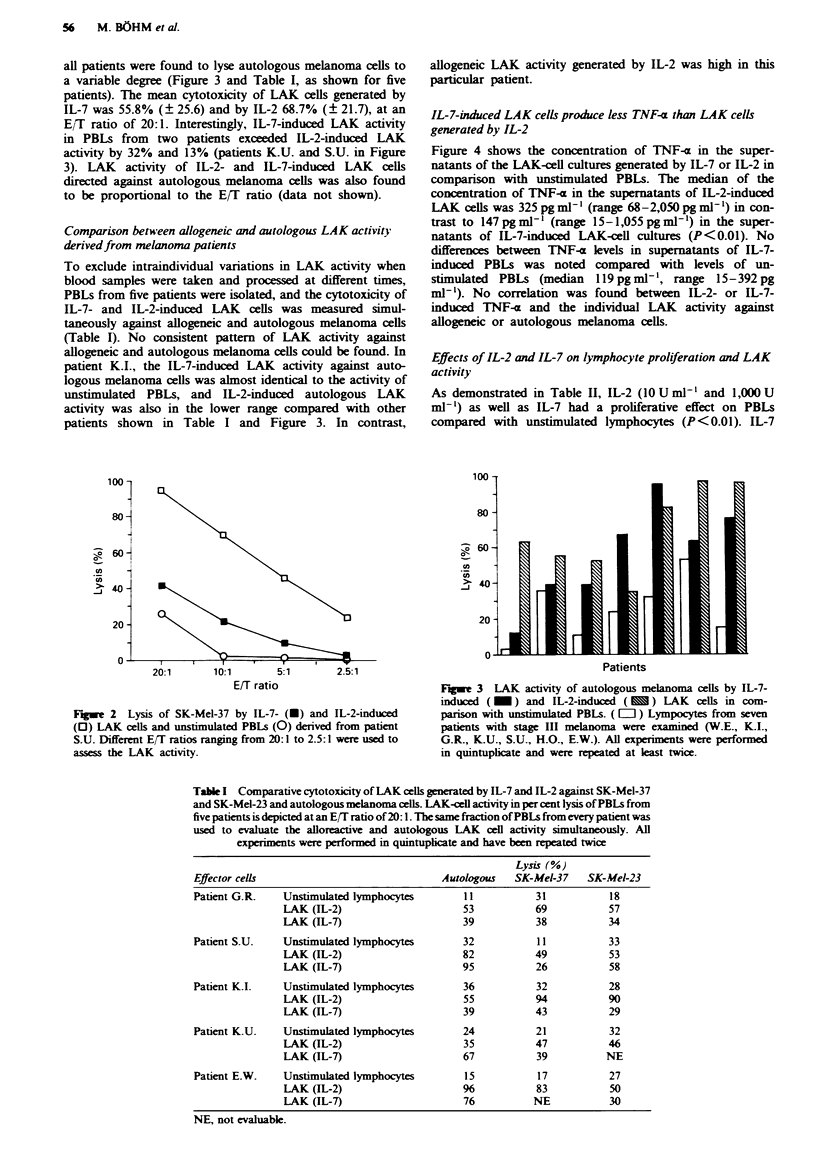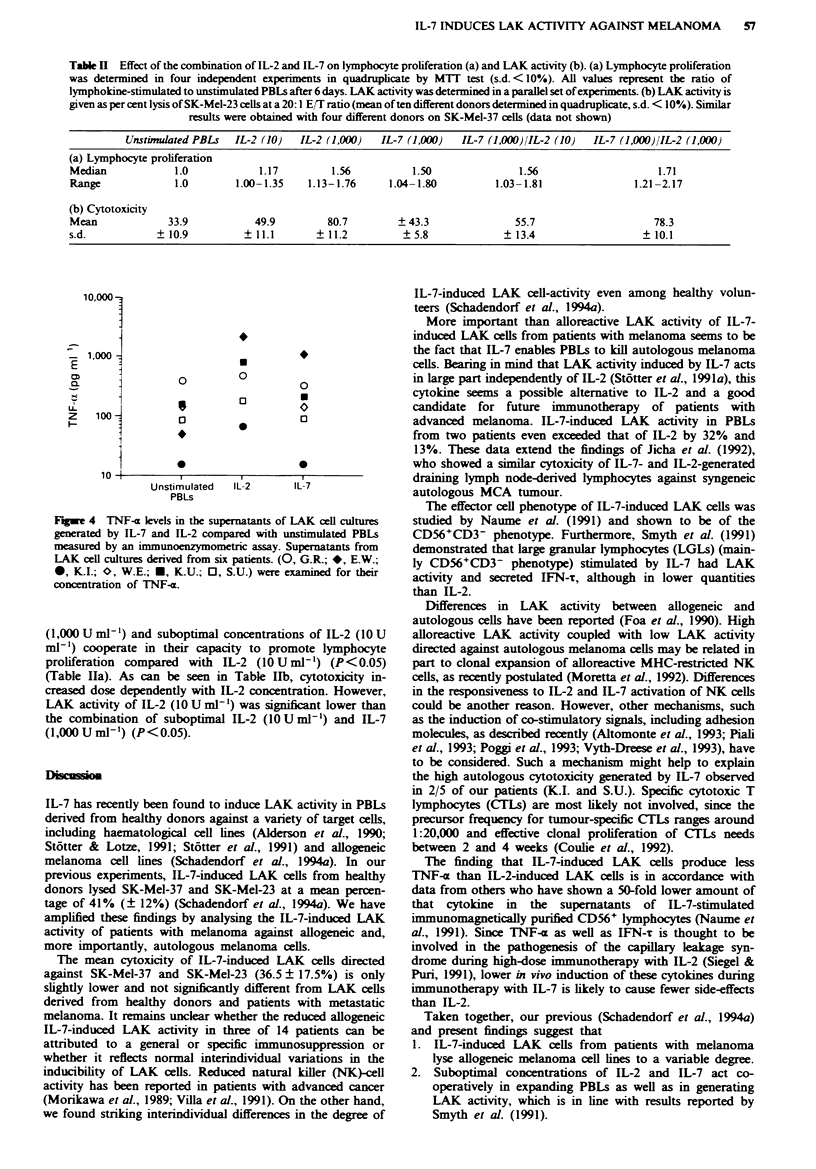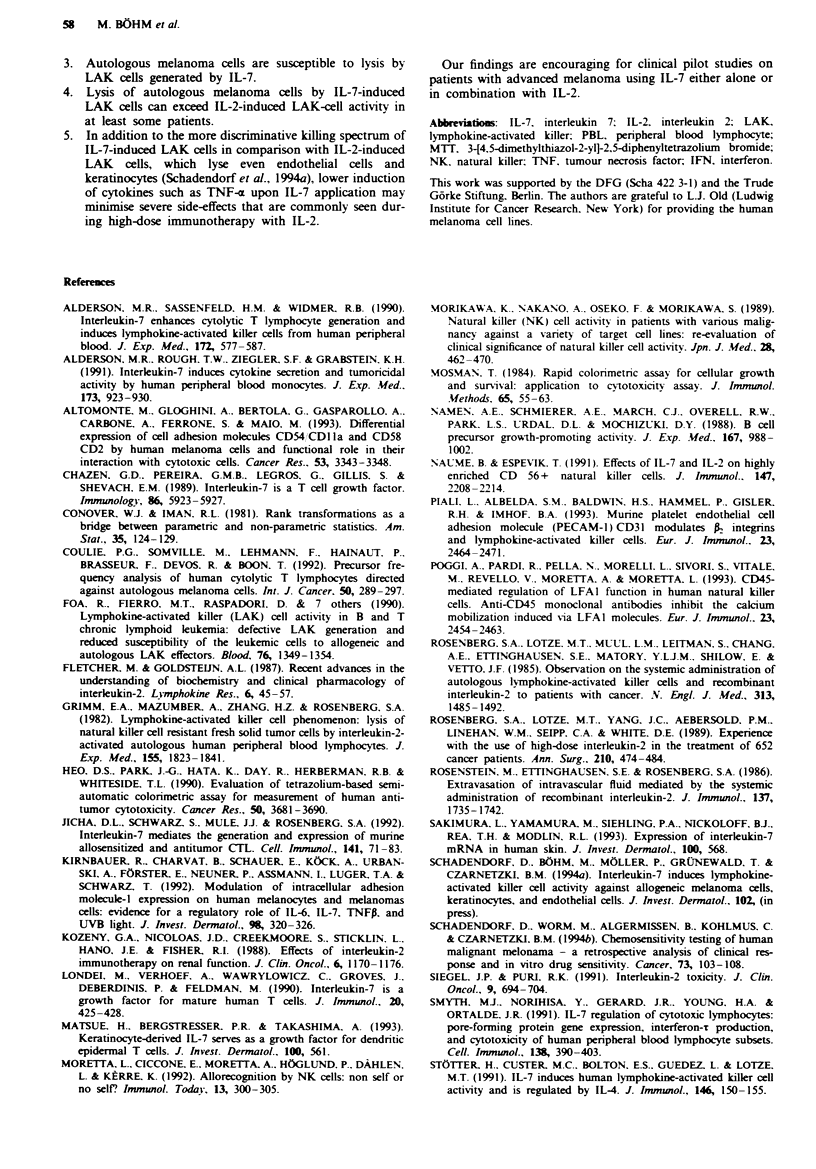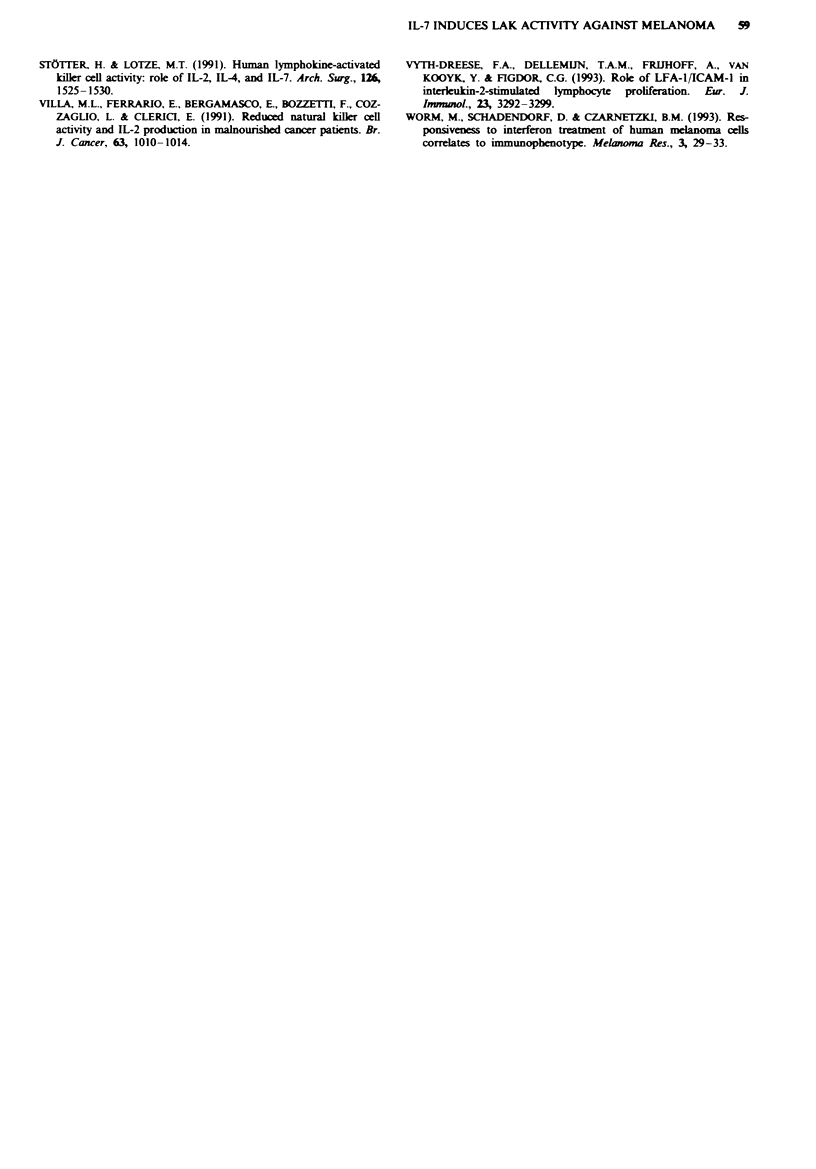# Lysis of allogeneic and autologous melanoma cells by IL-7-induced lymphokine-activated killer cells.

**DOI:** 10.1038/bjc.1994.249

**Published:** 1994-07

**Authors:** M. Böhm, P. Möller, U. Kalbfleisch, M. Worm, B. M. Czarnetzki, D. Schadendorf

**Affiliations:** University Hospital Rudolf Virchow, Department of Dermatology, Free University of Berlin, Germany.

## Abstract

In order to assess the potential of interleukin 7 (IL-7) as an immunotherapeutic agent in human melanoma, we have evaluated the in vitro activity of IL-7-induced lymphokine-activated killer (LAK) cells from patients with advanced melanoma against allogeneic and autologous melanoma cells. Peripheral blood lymphocytes (PBLs) from 14 patients with stage III melanoma were isolated and incubated in the presence of 1,000 U ml-1 IL-7 and 100 U ml-1 IL-2 for comparison. LAK-cell activity was determined by a 24 h cytotoxicity assay using MTT [3-(4,5-dimethylthiazol-2-yl)-2,5-diphenyltetrazolium bromide]. The activity of IL-7-induced LAK cells against two allogeneic melanoma cell lines was 32.7% (+/- 17.9) against SK-Mel-37 and 38.1% (+/- 12.5) against SK-Mel-23 at an effector-to-target (E/T) ratio of 20:1. The activity of IL-2-induced LAK cells was significantly higher against SK-Mel-37 (78 +/- 24.6%) and against SK-Mel-23 (73.5 +/- 19.7%). IL-7 and suboptimal doses of IL-2 (10 U ml-1) were found to have a co-stimulatory on lymphocyte proliferation as well as on LAK activity. Against autologous melanoma cells, the activity of IL-7- and IL-2-induced LAK cells did not differ significantly (55.8 +/- 25.6% versus 68.7 +/- 21.7% respectively). In two patients, IL-7-induced LAK-cell activity against autologous melanoma cells exceeded even that of IL-2 significantly (67% vs 35% and 95% vs 82%). Levels of tumour necrosis factor alpha (TNF-alpha) in the supernatants of LAK-cell cultures generated by IL-7 were lower than those of IL-2-generated LAK-cell cultures. These results suggest that IL-7 is a potential alternative to immunotherapy with IL-2 in terms of efficacy and possible side-effects and encourages pilot studies with IL-7 in melanoma patients.


Br. J. Cancer (1994), 70, 54–59 © Macmillan Press Ltd., 1994

### Lysis of allogeneic and autologous melanoma cells by IL-7-induced lymphokine-activated killer cells*

M. Böhm†, P. Möller, U. Kalbfleisch, M. Worm, B.M. Czarnetzki & D. Schadendorf

*University Hospital Rudolf Virchow, Department of Dermatology, Free University of Berlin, Germany.*

**Summary** In order to assess the potential of interleukin 7 (IL-7) as an immunotherapeutic agent in human melanoma, we have evaluated the *in vitro* activity of IL-7-induced lymphokine-activated killer (LAK) cells from patients with advanced melanoma against allogeneic and autologous melanoma cells. Peripheral blood lymphocytes (PBLs) from 14 patients with stage III melanoma were isolated and incubated in the presence of 1,000 U $ml^{-1}$ IL-7 and 100 U $ml^{-1}$ IL-2 for comparison. LAK-cell activity was determined by a 24 h cytotoxicity assay using MTT [3-(4,5-dimethylthiazol-2-yl)-2,5-diphenyltetrazolium bromide]. The activity of IL-7-induced LAK cells against two allogeneic melanoma cell lines was 32.7% ($\pm$ 17.9) against SK-Mel-37 and 38.1% ($\pm$ 12.5) against SK-Mel-23 at an effector-to-target (E/T) ratio of 20:1. The activity of IL-2-induced LAK cells was significantly higher against SK-Mel-37 (78 $\pm$ 24.6%) and against SK-Mel-23 (73.5 $\pm$ 19.7%). IL-7 and suboptimal doses of IL-2 (10 U $ml^{-1}$) were found to have a co-stimulatory on lymphocyte proliferation as well as on LAK activity. Against autologous melanoma cells, the activity of IL-7- and IL-2-induced LAK cells did not differ significantly (55.8 $\pm$ 25.6% versus 68.7 $\pm$ 21.7% respectively). In two patients, IL-7-induced LAK-cell activity against autologous melanoma cells exceeded even that of IL-2 significantly (67% *vs* 35% and 95% *vs* 82%). Levels of tumour necrosis factor α (TNF-α) in the supernatants of LAK-cell cultures generated by IL-7 were lower than those of IL-2-generated LAK-cell cultures. These results suggest that IL-7 is a potential alternative to immunotherapy with IL-2 in terms of efficacy and possible side-effects and encourages pilot studies with IL-7 in melanoma patients.

Immunotherapy with IL-2 with or without LAK cells has achieved some impressive regressions in patients with advanced melanoma who had failed on conventional therapy (Rosenberg *et al.*, 1985; Fletcher *et al.*, 1987). However, the clinical responses to this kind of therapy are infrequent and often transient (Rosenberg *et al.*, 1989). Furthermore, high-dose immunotherapy with IL-2 is associated with severe toxicity including pulmonary congestion, hypotension, oedema or anuria, known as the capillary leakage syndrome (Rosenstein *et al.*, 1986; Kozeny *et al.*, 1988). These limitations of an initially promising kind of immunotherapy have led to an extensive search for better-tolerated treatment schedules as well as alternative agents in immunotherapy of human melanoma. IL-7 was originally described as a growth factor for early B cells (Namen *et al.*, 1988). Further characterization revealed that IL-7 is also an important differentiation factor for the maturation and proliferation of T lymphocytes (Chazen *et al.*, 1989; Londei *et al.*, 1990). IL-7 supports the development of cytotoxic T cells and generates LAK activity in human PBLs against Daudi cells (Alderson *et al.*, 1990; Stötter & Lotze, 1991; Stötter *et al.*, 1991). Recent data indicate that the spectrum of cells responsive to IL-7 is broader than originally expected and includes monocytes (Alderson *et al.*, 1991) and melanoma cells (Kirnbauer *et al.*, 1992). Therefore, IL-7 can be considered to be a multifunctional cytokine acting on various cell lineages. Most recently, human keratinocytes have been shown to secrete IL-7 (Sakimura *et al.*, 1993), suggesting an important immunoregulatory role of this cytokine for dendritic T cells with in the epidermal compartment (Matsue *et al.*, 1993). We have recently shown that melanoma cells from allogeneic melanoma cell lines can be lysed by IL-7-induced LAK cells from healthy donors (Schadendorf *et al.*, 1994*a*). IL-7-generated LAK activity against allogeneic melanoma cell lines with early and late differentiation markers is weaker than IL-2-induced LAK-cell activity. However, sparing of keratinocytes and endothelial cells predicts that LAK cells generated by IL-7 are more discriminative in their target cell spectrum than IL-2-induced LAK cells (Schadendorf *et al.*, 1994*a*). In order to address the question of whether patients with advanced malignant melanoma could benefit from immunotherapy with IL-7, we have examined the *in vitro* susceptibility of allogeneic as well as autologous melanoma cells to LAK cells (from 14 patients with metastatic melanoma) stimulated with IL-7 or IL-2. In addition, we have evaluated the comparative concentrations of TNF-α in the supernatants of LAK-cell cultures generated by IL-7 or IL-2 since secondarily induced cytokines such as TNF-α are thought to play a key role in the development of side-effects during immunotherapy with IL-2 (Siegel & Puri, 1991).

### Material and methods

### *Cytokines*

Human recombinant IL-7 was provided by Immunex (Seattle, WA, USA) with a specific activity of $4.7 \times 10^7$ U $mg^{-1}$ and 3.9 endotoxin units per ng of protein. The specific activity was determined with a bioassay that employs the IL-7-dependent IxN/Lb pre-B-cell line (Namen *et al.*, 1988). Human recombinant IL-2 was purchased from Boehringer (Mannheim, Germany) and had a specific activity of $2 \times 10^6$ U $mg^{-1}$ and fewer than 100 endotoxin units per ng.

### *Cell culture of melanoma cell lines*

The human melanoma cell lines SK-Mel-37 and SK-Mel-23 were grown as previously described (Schadendorf *et al.*, 1994*b*). Both cell lines were established several years ago at the Memorial Sloan Kettering Cancer Center from metastatic lesions derived from patients with malignant melanoma. Their differentiation markers have been characterised extensively (Worm *et al.*, 1993).

### *Preparation of autologous melanoma cells*

Tumour specimens were collected from patients with advanced melanoma undergoing procedures as a part of either diagnostic work-up or treatment of their disease. Fresh

Correspondence: D. Schadendorf, Department of Dermatology, University Hospital Rudolf Virchow, Free University Berlin, Augustenburger Platz 1, 13344 Berlin, Germany.
*This work was in part presented at the Annual Meeting of the Society of Investigative Dermatology, 28 April to 1 May, 1993, Washington, USA
†Present address: Yale University School of Medicine, Department of Dermatology, New Haven, CT, USA.
Received 16 August 1993; and in revised form 17 February 1994.

melanoma cells from tumour specimens from lymph nodes, cutis or subcutis were prepared as described previously (Schadendorf *et al.*, 1994*b*). For testing, passages 2–5 were used. Parallel cytospins of the tumour cells were done, and it was confirmed by immunocytochemistry that >95% were S-100 positive.

*LAK-cell generation from patients with melanoma*

Peripheral blood mononuclear cells (PBMCs) were obtained from 14 patients with stage III melanoma (age 32–89, equal sex ratio) by Ficoll–Hypaque centrifugation. Any form of chemotherapy, immunotherapy or hormone therapy had to be discontinued 6 weeks before blood collection. After removal of the adherent monocytes by incubating the PBMCs on plastic plates for 1 h at 37°C, the PBLs were decanted and either cryopreserved or immediately processed for the generation of LAK cells (Grimm *et al.*, 1982; Schadendorf *et al.*, 1994*b*). In short, PBLs were incubated at a density of $2 \times 10^6$ cells $ml^{-1}$ in RPMI-1640 supplemented with glutamine, fetal calf serum and antibiotics as mentioned above, and 1,000 U $ml^{-1}$ IL-7 or 100 U $ml^{-1}$ IL-2. One thousand units of IL-7 represents 0.1 mg and 100 U of IL-2 represents 0.033 mg. The cells were incubated in 25 $cm^2$ flasks (Greiner, Frickenhausen, Germany) in an upright position for 4 days in 5% carbon dioxide at 37°C. On day 4, LAK-cell concentration was determined by haemocytometer counts of cells excluding 0.18% trypan blue dye.

*Cytotoxicity assay*

LAK-cell activity was determined with a 24 h tetrazolium-based colorimetric assay originally described by Mosman (1983). The measured absorbance is proportional to the number of viable cells. Modified as a cytotoxicity assay for the quantification of anti-tumour effects mediated by LAK cells, this test has been found to compare favourably with the $^{51}$Cr-release assay in terms of sensitivity and reproducibility, particularly if long-term cytotoxicity is to be measured (Heo *et al.*, 1990). The MTT assay was performed as follows. Melanoma cells were brought to single-cell suspension by incubation with 0.25% trypsin–EDTA solution (Seromed, Berlin, Germany). Cell concentration and viability were determined by haemocytometer counts excluding 0.18% trypan blue dye. Melanoma cells were plated at a density of $1 \times 10^4$ cells per well in 96-well flat-bottomed tissue culture microtitre plates (Greiner) 20 h prior to the addition of LAK cells. LAK cells were added at different E/T ratios ranging from 20:1 to 2.5:1. Quintuplicates were used for every E/T ratio. After incubation for 24 h, non-adherent cells were removed by performing three consecutive washings with unsupplemented medium. To ensure lethal damage of detached cells, recultivation of detached cells was performed, which failed in all experiments. MTT (Sigma, Deisenhofen, Germany) at a final concentration of 0.5 mg $ml^{-1}$ was added to each well and plates were incubated for 4 h at 37°C. Absorbance was read at a wavelength of 540 nm on a scanning multiwell spectrophotometer (Titertek Multiscan MCC/340, Meckenheim, Germany). The percentage of lysis was determined as:

$$\text{Lysis } (\%) = \frac{A - (B - C)}{A} \times 100$$

where $A$ is the mean optical density of $1 \times 10^4$ melanoma cells without addition of LAK cells, $B$ is the mean experimental absorbance of adherent melanoma cells remaining in the wells after washing and $C$ represents a correction factor for 'sticky' LAK cells that remain in the wells after washing and was obtained by plating different numbers of LAK cells in the wells without melanoma cells added.

*Determination of TNF-α*

TNF-α levels in the supernatants of LAK-cell cultures were measured by a quantitative immunoenzymometric kit (R&D Systems, Quantikine, Minneapolis, USA). The sensitivity was 4.8 pg $ml^{-1}$ as indicated by the manufacturer.

*Statistical analysis*

The statistical significance of the data obtained from the cytoxicity assays and TNF-τ levels was calculated using a modified Wilcoxon signed-rank test (Conover & Iman, 1981). The procedure involves conversion of the observations to rank order and subsequent calculation of the *t*-statistic by using ranks instead of the original observations.

### Results

*IL-7-induced LAK cells from patients with melanoma are cytotoxic against allogeneic melanoma cells*

IL-7-induced LAK activity against SK-Mel-37 and SK-Mel-23 was found to be higher than the cytotoxicity of unstimulated lymphocytes in 11 of 14 patients. The mean cytoxicity of LAK cells generated by IL-7 was 32.7% (± 17.9) against SK-Mel-37 and 38.1% (± 12.5) against SK-Mel-23 at an E/T ratio of 20:1 (Figure 1). Induction of IL-7-induced LAK activity was statistically significant compared with unstimulated lymphocytes ($P = 0.05$). In three out of 14 patients, IL-7-induced LAK activity against either SK-Mel-37 or SK-Mel-23 did not exceed that of unstimulated PBLs. However, the reduced IL-7-induced LAK activity in these patients did not correlate with age, sex or tumour burden. IL-7-induced LAK activity was also found to be proportional to the E/T ratio (Figure 2, as paradigmatically shown for one patient).

IL-2-induced LAK activity higher than activity of unstimulated PBLs was generated in 13 out of 14 patients and was higher than LAK activity generated by IL-7 in all patients (Figure 1). The mean cytotoxicity of IL-2-generated LAK cells was 78% (± 24.6) against SK-Mel-37 and 73.5% (± 19.7) against SK-Mel-23 at an E/T ratio of 20:1. No significant differences were found regarding the susceptibility of the highly differentiated melanoma cell line SK-Mel-23 and the lesser differentiated cell line SK-Mel-37 to either IL-7- or IL-2-induced LAK cells.

*IL-7 induces LAK activity against autologous melanoma cells*

LAK cells from seven patients were available for assessing their autologous LAK activity. IL-7-induced LAK cells from

**Figure 1** *In vitro* LAK activity of IL-7-induced (■) and IL-2-induced (▨) LAK cells derived from 14 patients with advanced melanoma. The percentage lysis of two allogeneic melanoma cells lines (SK-Mel-37 and SK-Mel-23) which differ in their differentiation features (Worm *et al.*, 1993) is depicted at an E/T ratio of 20:1. Unstimulated PBLs (□) were grown in complete medium containing 10% fetal calf serum and were used as controls ($P = 0.05$). The mean percentage lysis and the standard deviation are given. All experiments were performed in quintuplicate and were repeated at least twice.

all patients were found to lyse autologous melanoma cells to a variable degree (Figure 3 and Table I, as shown for five patients). The mean cytotoxicity of LAK cells generated by IL-7 was 55.8% (± 25.6) and by IL-2 68.7% (± 21.7), at an E/T ratio of 20:1. Interestingly, IL-7-induced LAK activity in PBLs from two patients exceeded IL-2-induced LAK activity by 32% and 13% (patients K.U. and S.U. in Figure 3). LAK activity of IL-2- and IL-7-induced LAK cells directed against autologous melanoma cells was also found to be proportional to the E/T ratio (data not shown).

*Comparison between allogeneic and autologous LAK activity derived from melanoma patients*

To exclude intraindividual variations in LAK activity when blood samples were taken and processed at different times, PBLs from five patients were isolated, and the cytotoxicity of IL-7- and IL-2-induced LAK cells was measured simultaneously against allogeneic and autologous melanoma cells (Table I). No consistent pattern of LAK activity against allogeneic and autologous melanoma cells could be found. In patient K.I., the IL-7-induced LAK activity against autologous melanoma cells was almost identical to the activity of unstimulated PBLs, and IL-2-induced autologous LAK activity was also in the lower range compared with other patients shown in Table I and Figure 3. In contrast, allogeneic LAK activity generated by IL-2 was high in this particular patient.

*IL-7-induced LAK cells produce less TNF-α than LAK cells generated by IL-2*

Figure 4 shows the concentration of TNF-α in the supernatants of the LAK-cell cultures generated by IL-7 or IL-2 in comparison with unstimulated PBLs. The median of the concentration of TNF-α in the supernatants of IL-2-induced LAK cells was 325 pg $ml^{-1}$ (range 68–2,050 pg $ml^{-1}$) in contrast to 147 pg $ml^{-1}$ (range 15–1,055 pg $ml^{-1}$) in the supernatants of IL-7-induced LAK-cell cultures ($P<0.01$). No differences between TNF-α levels in supernatants of IL-7-induced PBLs was noted compared with levels of unstimulated PBLs (median 119 pg $ml^{-1}$, range 15–392 pg $ml^{-1}$). No correlation was found between IL-2- or IL-7-induced TNF-α and the individual LAK activity against allogeneic or autologous melanoma cells.

*Effects of IL-2 and IL-7 on lymphocyte proliferation and LAK activity*

As demonstrated in Table II, IL-2 (10 U $ml^{-1}$ and 1,000 U $ml^{-1}$) as well as IL-7 had a proliferative effect on PBLs compared with unstimulated lymphocytes ($P<0.01$). IL-7

**Figure 2** Lysis of SK-Mel-37 by IL-7- (■) and IL-2-induced (□) LAK cells and unstimulated PBLs (O) derived from patient S.U. Different E/T ratios ranging from 20:1 to 2.5:1 were used to assess the LAK activity.

**Figure 3** LAK activity of autologous melanoma cells by IL-7-induced (■) and IL-2-induced (▨) LAK cells in comparison with unstimulated PBLs. (□) Lympocytes from seven patients with stage III melanoma were examined (W.E., K.I., G.R., K.U., S.U., H.O., E.W.). All experiments were performed in quintuplicate and were repeated at least twice.

**Table I** Comparative cytotoxicity of LAK cells generated by IL-7 and IL-2 against SK-Mel-37 and SK-Mel-23 and autologous melanoma cells. LAK-cell activity in per cent lysis of PBLs from five patients is depicted at an E/T ratio of 20:1. The same fraction of PBLs from every patient was used to evaluate the alloreactive and autologous LAK cell activity simultaneously. All experiments were performed in quintuplicate and have been repeated twice

| *Effector cells* | | *Autologous* | *Lysis (%)* *SK-Mel-37* | *SK-Mel-23* |
|---|---|---|---|---|
| Patient G.R. | Unstimulated lymphocytes | 11 | 31 | 18 |
| | LAK (IL-2) | 53 | 69 | 57 |
| | LAK (IL-7) | 39 | 38 | 34 |
| Patient S.U. | Unstimulated lymphocytes | 32 | 11 | 33 |
| | LAK (IL-2) | 82 | 49 | 53 |
| | LAK (IL-7) | 95 | 26 | 58 |
| Patient K.I. | Unstimulated lymphocytes | 36 | 32 | 28 |
| | LAK (IL-2) | 55 | 94 | 90 |
| | LAK (IL-7) | 39 | 43 | 29 |
| Patient K.U. | Unstimulated lymphocytes | 24 | 21 | 32 |
| | LAK (IL-2) | 35 | 47 | 46 |
| | LAK (IL-7) | 67 | 39 | NE |
| Patient E.W. | Unstimulated lymphocytes | 15 | 17 | 27 |
| | LAK (IL-2) | 96 | 83 | 50 |
| | LAK (IL-7) | 76 | NE | 30 |

NE, not evaluable.

**Table II** Effect of the combination of IL-2 and IL-7 on lymphocyte proliferation (a) and LAK activity (b). (a) Lymphocyte proliferation was determined in four independent experiments in quadruplicate by MTT test (s.d. < 10%). All values represent the ratio of lymphokine-stimulated to unstimulated PBLs after 6 days. LAK activity was determined in a parallel set of experiments. (b) LAK activity is given as per cent lysis of SK-Mel-23 cells at a 20:1 E/T ratio (mean of ten different donors determined in quadruplicate, s.d. < 10%). Similar results were obtained with four different donors on SK-Mel-37 cells (data not shown)

| | *Unstimulated PBLs* | *IL-2 (10)* | *IL-2 (1,000)* | *IL-7 (1,000)* | *IL-7 (1,000)/IL-2 (10)* | *IL-7 (1,000)/IL-2 (1,000)* |
|---|---|---|---|---|---|---|
| (a) Lymphocyte proliferation | | | | | | |
| Median | 1.0 | 1.17 | 1.56 | 1.50 | 1.56 | 1.71 |
| Range | 1.0 | 1.00–1.35 | 1.13–1.76 | 1.04–1.80 | 1.03–1.81 | 1.21–2.17 |
| (b) Cytotoxicity | | | | | | |
| Mean | 33.9 | 49.9 | 80.7 | ± 43.3 | 55.7 | 78.3 |
| s.d. | ± 10.9 | ± 11.1 | ± 11.2 | ± 5.8 | ± 13.4 | ± 10.1 |

**Figure 4** TNF-α levels in the supernatants of LAK cell cultures generated by IL-7 and IL-2 compared with unstimulated PBLs measured by an immunoenzymometric assay. Supernatants from LAK cell cultures derived from six patients. (○, G.R.; ◆, E.W.; ●, K.I.; ◇, W.E.; ■, K.U.; □, S.U.) were examined for their concentration of TNF-α.

(1,000 U $ml^{-1}$) and suboptimal concentrations of IL-2 (10 U $ml^{-1}$) cooperate in their capacity to promote lymphocyte proliferation compared with IL-2 (10 U $ml^{-1}$) ($P<0.05$) (Table IIa). As can be seen in Table IIb, cytotoxicity increased dose dependently with IL-2 concentration. However, LAK activity of IL-2 (10 U $ml^{-1}$) was significant lower than the combination of suboptimal IL-2 (10 U $ml^{-1}$) and IL-7 (1,000 U $ml^{-1}$) ($P<0.05$).

### Discussion

IL-7 has recently been found to induce LAK activity in PBLs derived from healthy donors against a variety of target cells, including haematological cell lines (Alderson *et al.*, 1990; Stötter & Lotze, 1991; Stötter *et al.*, 1991) and allogeneic melanoma cell lines (Schadendorf *et al.*, 1994*a*). In our previous experiments, IL-7-induced LAK cells from healthy donors lysed SK-Mel-37 and SK-Mel-23 at a mean percentage of 41% (± 12%) (Schadendorf *et al.*, 1994*a*). We have amplified these findings by analysing the IL-7-induced LAK activity of patients with melanoma against allogeneic and, more importantly, autologous melanoma cells.

The mean cytoxicity of IL-7-induced LAK cells directed against SK-Mel-37 and SK-Mel-23 (36.5 ± 17.5%) is only slightly lower and not significantly different from LAK cells derived from healthy donors and patients with metastatic melanoma. It remains unclear whether the reduced allogeneic IL-7-induced LAK activity in three of 14 patients can be attributed to a general or specific immunosuppression or whether it reflects normal interindividual variations in the inducibility of LAK cells. Reduced natural killer (NK)-cell activity has been reported in patients with advanced cancer (Morikawa *et al.*, 1989; Villa *et al.*, 1991). On the other hand, we found striking interindividual differences in the degree of IL-7-induced LAK cell-activity even among healthy volunteers (Schadendorf *et al.*, 1994*a*).

More important than alloreactive LAK activity of IL-7-induced LAK cells from patients with melanoma seems to be the fact that IL-7 enables PBLs to kill autologous melanoma cells. Bearing in mind that LAK activity induced by IL-7 acts in large part independently of IL-2 (Stötter *et al.*, 1991*a*), this cytokine seems a possible alternative to IL-2 and a good candidate for future immunotherapy of patients with advanced melanoma. IL-7-induced LAK activity in PBLs from two patients even exceeded that of IL-2 by 32% and 13%. These data extend the findings of Jicha *et al.* (1992), who showed a similar cytoxicity of IL-7- and IL-2-generated draining lymph node-derived lymphocytes against syngeneic autologous MCA tumour.

The effector cell phenotype of IL-7-induced LAK cells was studied by Naume *et al.* (1991) and shown to be of the $CD56^+CD3^-$ phenotype. Furthermore, Smyth *et al.* (1991) demonstrated that large granular lymphocytes (LGLs) (mainly $CD56^+CD3^-$ phenotype) stimulated by IL-7 had LAK activity and secreted IFN-τ, although in lower quantities than IL-2.

Differences in LAK activity between allogeneic and autologous cells have been reported (Foa *et al.*, 1990). High alloreactive LAK activity coupled with low LAK activity directed against autologous melanoma cells may be related in part to clonal expansion of alloreactive MHC-restricted NK cells, as recently postulated (Moretta *et al.*, 1992). Differences in the responsiveness to IL-2 and IL-7 activation of NK cells could be another reason. However, other mechanisms, such as the induction of co-stimulatory signals, including adhesion molecules, as described recently (Altomonte *et al.*, 1993; Piali *et al.*, 1993; Poggi *et al.*, 1993; Vyth-Dreese *et al.*, 1993), have to be considered. Such a mechanism might help to explain the high autologous cytotoxicity generated by IL-7 observed in 2/5 of our patients (K.I. and S.U.). Specific cytotoxic T lymphocytes (CTLs) are most likely not involved, since the precursor frequency for tumour-specific CTLs ranges around 1:20,000 and effective clonal proliferation of CTLs needs between 2 and 4 weeks (Coulie *et al.*, 1992).

The finding that IL-7-induced LAK cells produce less TNF-α than IL-2-induced LAK cells is in accordance with data from others who have shown a 50-fold lower amount of that cytokine in the supernatants of IL-7-stimulated immunomagnetically purified $CD56^+$ lymphocytes (Naume *et al.*, 1991). Since TNF-α as well as IFN-τ is thought to be involved in the pathogenesis of the capillary leakage syndrome during high-dose immunotherapy with IL-2 (Siegel & Puri, 1991), lower *in vivo* induction of these cytokines during immunotherapy with IL-7 is likely to cause fewer side-effects than IL-2.

Taken together, our previous (Schadendorf *et al.*, 1994*a*) and present findings suggest that

1. IL-7-induced LAK cells from patients with melanoma lyse allogeneic melanoma cell lines to a variable degree.
2. Suboptimal concentrations of IL-2 and IL-7 act co-operatively in expanding PBLs as well as in generating LAK activity, which is in line with results reported by Smyth *et al.* (1991).

3. Autologous melanoma cells are susceptible to lysis by LAK cells generated by IL-7.
4. Lysis of autologous melanoma cells by IL-7-induced LAK cells can exceed IL-2-induced LAK-cell activity in at least some patients.
5. In addition to the more discriminative killing spectrum of IL-7-induced LAK cells in comparison with IL-2-induced LAK cells, which lyse even endothelial cells and keratinocytes (Schadendorf et al., 1994a), lower induction of cytokines such as TNF-$\alpha$ upon IL-7 application may minimise severe side-effects that are commonly seen during high-dose immunotherapy with IL-2.

Our findings are encouraging for clinical pilot studies on patients with advanced melanoma using IL-7 either alone or in combination with IL-2.

**Abbreviations**: IL-7, interleukin 7; IL-2, interleukin 2; LAK, lymphokine-activated killer; PBL, peripheral blood lymphocyte; MTT, 3-[4,5-dimethylthiazol-2-yl]-2,5-diphenyltetrazolium bromide; NK, natural killer; TNF, tumour necrosis factor; IFN, interferon.

This work was supported by the DFG (Scha 422 3-1) and the Trude Görke Stiftung, Berlin. The authors are grateful to L.J. Old (Ludwig Institute for Cancer Research, New York) for providing the human melanoma cell lines.

**References**

ALDERSON, M.R., SASSENFELD, H.M. & WIDMER, R.B. (1990). Interleukin-7 enhances cytolytic T lymphocyte generation and induces lymphokine-activated killer cells from human peripheral blood. *J. Exp. Med.*, **172**, 577-587.

ALDERSON, M.R., ROUGH, T.W., ZIEGLER, S.F. & GRABSTEIN, K.H. (1991). Interleukin-7 induces cytokine secretion and tumoricidal activity by human peripheral blood monocytes. *J. Exp. Med.*, **173**, 923-930.

ALTOMONTE, M., GLOGHINI, A., BERTOLA, G., GASPAROLLO, A., CARBONE, A., FERRONE, S. & MAIO, M. (1993). Differential expression of cell adhesion molecules CD54/CD11a and CD58/CD2 by human melanoma cells and functional role in their interaction with cytotoxic cells. *Cancer Res.*, **53**, 3343-3348.

CHAZEN, G.D., PEREIRA, G.M.B., LEGROS, G., GILLIS, S. & SHEVACH, E.M. (1989). Interleukin-7 is a T cell growth factor. *Immunology*, **86**, 5923-5927.

CONOVER, W.J. & IMAN, R.L. (1981). Rank transformations as a bridge between parametric and non-parametric statistics. *Am. Stat.*, **35**, 124-129.

COULIE, P.G., SOMVILLE, M., LEHMANN, F., HAINAUT, P., BRASSEUR, F., DEVOS, R. & BOON, T. (1992). Precursor frequency analysis of human cytolytic T lymphocytes directed against autologous melanoma cells. *Int. J. Cancer*, **50**, 289-297.

FOA, R., FIERRO, M.T., RASPADORI, D. & 7 others (1990). Lymphokine-activated killer (LAK) cell activity in B and T chronic lymphoid leukemia: defective LAK generation and reduced susceptibility of the leukemic cells to allogeneic and autologous LAK effectors. *Blood*, **76**, 1349-1354.

FLETCHER, M. & GOLDSTEIJN, A.L. (1987). Recent advances in the understanding of biochemistry and clinical pharmacology of interleukin-2. *Lymphokine Res.*, **6**, 45-57.

GRIMM, E.A., MAZUMBER, A., ZHANG, H.Z. & ROSENBERG, S.A. (1982). Lymphokine-activated killer cell phenomenon: lysis of natural killer cell resistant fresh solid tumor cells by interleukin-2-activated autologous human peripheral blood lymphocytes. *J. Exp. Med.*, **155**, 1823-1841.

HEO, D.S., PARK, J.-G., HATA, K., DAY, R., HERBERMAN, R.B. & WHITESIDE, T.L. (1990). Evaluation of tetrazolium-based semiautomatic colorimetric assay for measurement of human antitumor cytotoxicity. *Cancer Res.*, **50**, 3681-3690.

JICHA, D.L., SCHWARZ, S., MULE, J.J. & ROSENBERG, S.A. (1992). Interleukin-7 mediates the generation and expression of murine allosensitized and antitumor CTL. *Cell. Immunol.*, **141**, 71-83.

KIRNBAUER, R., CHARVAT, B., SCHAUER, E., KÖCK, A., URBANSKI, A., FÖRSTER, E., NEUNER, P., ASSMANN, I., LUGER, T.A. & SCHWARZ, T. (1992). Modulation of intracellular adhesion molecule-1 expression on human melanocytes and melanomas cells: evidence for a regulatory role of IL-6, IL-7, TNF$\beta$, and UVB light. *J. Invest. Dermatol.*, **98**, 320-326.

KOZENY, G.A., NICOLOAS, J.D., CREEKMOORE, S., STICKLIN, L., HANO, J.E. & FISHER, R.I. (1988). Effects of interleukin-2 immunotherapy on renal function. *J. Clin. Oncol.*, **6**, 1170-1176.

LONDEI, M., VERHOEF, A., WAWRYLOWICZ, C., GROVES, J., DEBERDINIS, P. & FELDMAN, M. (1990). Interleukin-7 is a growth factor for mature human T cells. *J. Immunol.*, **20**, 425-428.

MATSUE, H., BERGSTRESSER, P.R. & TAKASHIMA, A. (1993). Keratinocyte-derived IL-7 serves as a growth factor for dendritic epidermal T cells. *J. Invest. Dermatol.*, **100**, 561.

MORETTA, L., CICCONE, E., MORETTA, A., HÖGLUND, P., DÅHLEN, L. & KÈRRE, K. (1992). Allorecognition by NK cells: non self or no self? *Immunol. Today*, **13**, 300-305.

MORIKAWA, K., NAKANO, A., OSEKO, F. & MORIKAWA, S. (1989). Natural killer (NK) cell activity in patients with various malignancy against a variety of target cell lines: re-evaluation of clinical significance of natural killer cell activity. *Jpn. J. Med.*, **28**, 462-470.

MOSMAN, T. (1984). Rapid colorimetric assay for cellular growth and survival: application to cytotoxicity assay. *J. Immunol. Methods*, **65**, 55-63.

NAMEN, A.E., SCHMIERER, A.E., MARCH, C.J., OVERELL, R.W., PARK, L.S., URDAL, D.L. & MOCHIZUKI, D.Y. (1988). B cell precursor growth-promoting activity. *J. Exp. Med.*, **167**, 988-1002.

NAUME, B. & ESPEVIK, T. (1991). Effects of IL-7 and IL-2 on highly enriched CD 56+ natural killer cells. *J. Immunol.*, **147**, 2208-2214.

PIALI, L., ALBELDA, S.M., BALDWIN, H.S., HAMMEL, P., GISLER, R.H. & IMHOF, B.A. (1993). Murine platelet endothelial cell adhesion molecule (PECAM-1) CD31 modulates $\beta_2$ integrins and lymphokine-activated killer cells. *Eur. J. Immunol.*, **23**, 2464-2471.

POGGI, A., PARDI, R., PELLA, N., MORELLI, L., SIVORI, S., VITALE, M., REVELLO, V., MORETTA, A. & MORETTA, L. (1993). CD45-mediated regulation of LFA1 function in human natural killer cells. Anti-CD45 monoclonal antibodies inhibit the calcium mobilization induced via LFA1 molecules. *Eur. J. Immunol.*, **23**, 2454-2463.

ROSENBERG, S.A., LOTZE, M.T., MUUL, L.M., LEITMAN, S., CHANG, A.E., ETTINGHAUSEN, S.E., MATORY, Y.L.J.M., SHILOW, E. & VETTO, J.F. (1985). Observation on the systemic administration of autologous lymphokine-activated killer cells and recombinant interleukin-2 to patients with cancer. *N. Engl. J. Med.*, **313**, 1485-1492.

ROSENBERG, S.A., LOTZE, M.T., YANG, J.C., AEBERSOLD, P.M., LINEHAN, W.M., SEIPP, C.A. & WHITE, D.E. (1989). Experience with the use of high-dose interleukin-2 in the treatment of 652 cancer patients. *Ann. Surg.*, **210**, 474-484.

ROSENSTEIN, M., ETTINGHAUSEN, S.E. & ROSENBERG, S.A. (1986). Extravasation of intravascular fluid mediated by the systemic administration of recombinant interleukin-2. *J. Immunol.*, **137**, 1735-1742.

SAKIMURA, L., YAMAMURA, M., SIEHLING, P.A., NICKOLOFF, B.J., REA, T.H. & MODLIN, R.L. (1993). Expression of interleukin-7 mRNA in human skin. *J. Invest. Dermatol.*, **100**, 568.

SCHADENDORF, D., BÖHM, M., MÖLLER, P., GRÜNEWALD, T. & CZARNETZKI, B.M. (1994a). Interleukin-7 induces lymphokine-activated killer cell activity against allogeneic melanoma cells, keratinocytes, and endothelial cells. *J. Invest. Dermatol.*, **102**, (in press).

SCHADENDORF, D., WORM, M., ALGERMISSEN, B., KOHLMUS, C. & CZARNETZKI, B.M. (1994b). Chemosensitivity testing of human malignant melonama - a retrospective analysis of clinical response and in vitro drug sensitivity. *Cancer*, **73**, 103-108.

SIEGEL, J.P. & PURI, R.K. (1991). Interleukin-2 toxicity. *J. Clin. Oncol.*, **9**, 694-704.

SMYTH, M.J., NORIHISA, Y., GERARD, J.R., YOUNG, H.A. & ORTALDE, J.R. (1991). IL-7 regulation of cytotoxic lymphocytes: pore-forming protein gene expression, interferon-$\tau$ production, and cytotoxicity of human peripheral blood lymphocyte subsets. *Cell. Immunol.*, **138**, 390-403.

STÖTTER, H., CUSTER, M.C., BOLTON, E.S., GUEDEZ, L. & LOTZE, M.T. (1991). IL-7 induces human lymphokine-activated killer cell activity and is regulated by IL-4. *J. Immunol.*, **146**, 150-155.

STÖTTER, H. & LOTZE, M.T. (1991). Human lymphokine-activated killer cell activity: role of IL-2, IL-4, and IL-7. *Arch. Surg.*, **126**, 1525–1530.

VILLA, M.L., FERRARIO, E., BERGAMASCO, E., BOZZETTI, F., COZZAGLIO, L. & CLERICI, E. (1991). Reduced natural killer cell activity and IL-2 production in malnourished cancer patients. *Br. J. Cancer*, **63**, 1010–1014.

VYTH-DREESE, F.A., DELLEMIJN, T.A.M., FRIJHOFF, A., VAN KOOYK, Y. & FIGDOR, C.G. (1993). Role of LFA-1/ICAM-1 in interleukin-2-stimulated lymphocyte proliferation. *Eur. J. Immunol.*, **23**, 3292–3299.

WORM, M., SCHADENDORF, D. & CZARNETZKI, B.M. (1993). Responsiveness to interferon treatment of human melanoma cells correlates to immunophenotype. *Melanoma Res.*, **3**, 29–33.